# A cross-sectional study assessing Australian general practice patients’ intention, reasons and preferences for assistance with losing weight

**DOI:** 10.1186/1471-2296-14-187

**Published:** 2013-12-10

**Authors:** Sze Lin Yoong, Mariko Leanne Carey, Robert William Sanson-Fisher, Catherine Anne D’Este

**Affiliations:** 1 Priority Research Centre for Health Behaviour, School of Medicine and Public Health, University of Newcastle, W4 Hunter Medical Research Institute, University of Newcastle, Newcastle 2308, Callaghan, Australia; 2 Hunter Medical Research Institute, 1 Kookaburra Circuit, New Lambton Hights 2305, Australia

**Keywords:** Obesity, Weight management, Family practice

## Abstract

**Background:**

The high prevalence of overweight and obesity in the population is concerning, as these conditions increase an individual’s risk of various chronic diseases. General practice is an ideal setting to target the reduction of overweight or obesity. Examining general practice patients’ intentions to lose weight and preferences for assistance with managing their weight is likely to be useful in informing weight management care provided in this setting. Thus, this study aimed to: 1) identify the proportion and characteristics of patients intending to change weight in the next six months; 2) reasons for intending to change weight and preferences for different modes of weight management assistance in overweight and obese patients.

**Methods:**

A cross-sectional study was conducted with 1,306 Australian adult general practice patients. Consenting patients reported via a touchscreen computer questionnaire their demographic characteristics, intention to lose weight in the next six months, reasons for wanting to lose weight, preferred personnel to assist with weight loss and willingness to accept support delivered via telephone, mobile and internet.

**Results:**

Fifty six percent (*n* = 731) of patients intended to lose weight in the next six months. Females, younger patients, those with a level of education of trade certificate and above or those with high cholesterol had significantly higher odds of intending to lose weight. “Health” was the top reason for wanting to lose weight in normal weight (38%), overweight (57%) and obese (72%) patients. More than half of overweight (61%) or obese (74%) patients reported that they would like help to lose weight from one of the listed personnel, with the dietitian and general practitioner (GP) being the most frequently endorsed person to help patients with losing weight. Almost 90% of overweight or obese participants indicated being willing to accept support with managing their weight delivered via the telephone.

**Conclusions:**

Most overweight or obese general practice patients intended to lose their weight in the next six months for health reasons. Younger females, with higher level of education or had high cholesterol had significantly higher odds of reporting intending to lose weight in the next six months. An opportunity exists for GPs to engage patients in weight loss discussions in the context of improving health. Interventions involving GP and dietitians with weight management support delivered via telephone, should be explored in future studies in this setting.

## Background

Overweight and obesity are modifiable risk factors for a range of chronic diseases and are highly prevalent conditions in developed countries including Australia [1] and United States (US) [2]. General practice represents a promising setting to target the management of excess weight. A large proportion of the population see their GP at least once a year and of those presenting for care, approximately 60% are overweight or obese [3]. Primary care guidelines recommend that GPs assess for overweight or obesity and initiate high intensity counselling and behavioural interventions for those overweight or obese [4,5]. Despite this little is known about the intentions, preferences and acceptability of weight management interventions amongst overweight or obese primary care patients. Previous studies have reported that characteristics such as sex, age, body mass index (BMI), ethnicity, socioeconomic status, number of physician visits and presence of chronic conditions are associated with current or previous attempts to lose weight [6,7]. There is, however, limited literature examining demographic and clinical characteristics associated with intentions to lose weight. Identifying the proportion and characteristics of those intending to lose weight is important to provide GPs with an indication of which patients are most likely to be responsive to discussions about weight loss strategies.

A patient-centred approach is recommended for all areas of health care. This involves provision of care, which is responsive to the needs, values and preferences of the patient [8]. Therefore, identifying what motivates patients to want to lose weight is likely to be important in informing the delivery of patient-centred weight management. Previous studies, conducted with participants enrolled in weight loss trial, have identified improving health and appearance as main motivators for wanting to lose weight in those overweight or obese [9-12]. In contrast to those recruited into weight loss trials with strict eligibility criteria, general practice patients presenting for care are likely to be a more heterogenous population, with different levels of motivation to change their weight. Further, those with elevated cardiovascular health problems are also likely to be excluded from clinical weight loss trials [13]. Hence data on the acceptability and preferences for intervention derived from such trials may not be generalisable to all patients in the primary care setting. While weight loss is not recommended for those of normal weight, a substantial proportion of those who are normal weight still report trying to lose weight [14]. Examining reasons for wanting to lose weight in normal weight patients can inform overall weight management discussions in this setting.

Although GPs play an important role in the overall management of overweight and obesity, they lack the time in a busy clinical setting to deliver high intensity interventions that are potentially effective in producing weight loss [15]. As such, the involvement of non-physician personnel or delivery of interventions via different modes may represent a promising way of providing these high intensity interventions to overweight or obese general practice patients [15]. Different types of health care professionals may provide different types of assistance with weight loss, and this may have implications for the acceptability of referrals. Thus, examining the types of personnel that patients would like help from in order to lose weight is crucial to maximise patient uptake of referrals and adherence to recommended strategies.

A number of interventions examining mobile phone, web-based and telephone delivery of weight loss support have reported promising outcomes [16-18]. With the increasing use of these technologies, examining patients’ willingness to accept support delivered via these mediums can provide an indication of the potential uptake of these types of interventions and inform the development of cost-effective interventions.

Therefore, this study aimed to examine the proportion of general practice patients who intended to lose weight in the next six months, demographic and clinical characteristics associated with intention to lose weight and reasons for wanting to lose weight. In overweight or obese patients who indicated intending to lose weight, preferred personnel to assist with weight management and willingness to accept weight management support delivered via telephone, mobile and internet were examined.

## Methods

### Study design

This cross-sectional study was conducted as part of larger study testing the feasibility of using a touchscreen computer health assessment in general practice patients.

### Practice recruitment

The sampling approach is described in detail elsewhere [19]. In summary, practices with more than two full time equivalent GPs and located within 20 km from a university department of general practice within the cities of Newcastle, Sydney and Melbourne were approached.

### Patient recruitment

Participants were adult general practice patients aged 18 years and older and judged by the research assistant (RA) as being physically and mentally able to provide informed consent. Patients who were pregnant were excluded from completing the survey assessing weight management practices.

### Procedure

The RA approached eligible patients in the waiting room about the study. Consenting patients completed a questionnaire administered using a portable touchscreen computer, while waiting for their general practice appointment. Patients were able to exit the survey if they were called in for their appointment. The sex of all approached patients was recorded by the RA on a log sheet.

### Equipment

Digivey Survey Suite software (CREOSO - Digivey Survey Center, Phoenix, Arizona) was used to program the patient survey. The survey was administered using Dell Latitude XT2 touchscreen laptop computers.

#### Measures

The questionnaire was pilot tested with behavioural researchers and 30 general practice patients (see Additional file 1 for questionnaire).

### Demographics

Participants provided information on their age, sex, ethnicity, level of education.

### Presence of weight–related chronic conditions

Patients reported whether they had high blood pressure, high cholesterol, heart problems, high blood sugar/glucose or chronic pain.

### Sufficient physical activity to meet guidelines

A one-item questionnaire was used to assess whether patients undertook at least half an hour of moderate or vigorous exercise on five or more days a week”. This tool has been shown to have 77% sensitivity and 81% specificity when compared to the New Zealand Physical Activity Questionnaire-Long Form [20]. Participants were classified as having insufficient levels of physical activity to meet guidelines if they indicated ‘no’ or ‘not sure’ on the above question.

### Smoking status

Participants were asked to report their current smoking status [21] and were categorised as being current smokers if they indicated smoking daily or smoking occasionally.

### Depression

The Patient Health Questionnaire-9 (PHQ-9) was used to assess depression. Those who scored ≥10 on this scale were categorised as being clinically depressed [22].

### Number of times seen GP

Patients were asked whether they were presenting to their usual GPs and the number of time they had seen the GP in the past 12 months.

### Weight and height

Participants were asked to report their weight in kilograms (kg) or stones and height in feet/inches or centimetres. Body Mass Index (BMI) was calculated using weight in kilograms (kg) divided by height in metres squared (m^2^). Participants were categorised as underweight if they had a BMI <18.5 kg/m^2^; normal weight if they had BMI between 18.5- 24.9 kg/m^2^; overweight if they had a BMI between 25–29.9 kg/m^2^ or obese if they had a BMI ≥ 30 kg/m^2^[23].

### Intention to change weight in next six months

Participants asked whether they intended to change their weight in the next six months. Response options were “Yes, intend to put on weight”, “Yes, intend to lose weight”, “No, do not intend to change weight” and “Not sure”. The following description was also included with the question “Intending to change weight in this question means that you have considered the benefits and costs of changing your weight. You are planning to make the required changes in the next 6 months in order to achieve this”.

### Reasons for weight loss

Patients who indicated intending to lose weight were asked to rank their top three reasons for wanting to do so. A review of the literature was carried out to identify potential reasons for intending to change weight. The response options included: “health reasons”, “to improve my appearance”, “to increase my confidence”, “to increase my physical fitness”, “to achieve my ideal weight”, “currently overweight”, “to feel better”; “to fit into my old clothes” and “other”.

### Preferences for professional assistance with weight loss

Patients who indicated intending to lose weight in the next six months were also asked to rank in order of preference which of the listed personnel they would like help from in order to change their weight. Response options included “general practitioner”, “practice nurse”, “dietitian”, “psychologist”, “exercise physiologist”, “surgeon”, “weight loss consultant” and “none of the above”.

### Willingness to accept support from different medium deliveries

Participants who indicated intending to lose weight in the next six months were asked if they were willing to accepted support with weight management via: a) telephone ; b) email; c) short messaging service (SMS); d) a smart phone /tablet application; e) online chat”. Participants could choose “Yes”, “No” or “No access”.

### Ethical approval

Ethical approval for this project was provided by the University of Newcastle Human Research Ethics Committee (HREC) (Approval no: HREC-2009-0341) and ratified by the University of New South Wales (Approval no: HREC 09393/ UN H-2009-0341) and Monash University HREC (2009001860).

#### Statistical analyses

Differences in sex of consenters and non-consenters were compared using Pearson’s Chi squared test. Those with a self-reported weight of less than 30 kg and more than 300 kg and/or a self-reported height of less than 120 cm and more than 250 cm were excluded from analyses as these values were perceived to be unrealistic. Those in the underweight group were excluded as there were only a small proportion of patients in this group. The demographic and clinical characteristics of normal weight, overweight and obese participants were reported and compared using a Chi-square test. The percentage of respondents indicating that they wanted to change their weight in the next six months was reported with 95% confidence interval (CI). Chi square tests were used to investigate the relationship between reporting intending to lose weight and age (18–24 years, 25–44 years, 45–65, ≥65 years), sex (male, female) , race (Caucasian/non-Caucasian), education (HSC and below, TAFE and Diploma, Tertiary, Postgraduate); exercise (met guidelines/did not meet guidelines); smoking (current smoker/not current smoker); depression (PHQ score <10 /PHQ score ≥10); number of time seen GP in last 12 months (three or less times, four to six times, seven to 10 times or > 10 times); presence of chronic pain, stroke, heart disease, high blood pressure, high cholesterol and type 2 diabetes (yes/no). Age was categorised to more closely match the Bettering the Evaluation and Care of Health Study (BEACH) study, an Australian longitudinal study conducted in general practice [24]. Variables with a p-value of less than 0.25 in the univariate analyses were included in a backward stepwise multiple logistic regression analysis and variables with a p-value of >0.1 on the adjusted Wald test were removed. Odds ratios, 95% CIs and p-values from the multiple logistic regression test variables included in the final model are reported. The number, proportion and 95% CIs endorsing each reason as one of their top or within top three reasons, their preferred personnel to help with losing weight as well as willingness to accept support delivered via different mediums were reported separately for normal weight, overweight or obese general practice patients and compared using Chi-square tests.

All 95% CIs and Chi square tests were adjusted for clustering of individuals within practices using svy, with the jackknife variance option. Statistical analysis was performed using STATA 11.0 (StataCorp LP, College Station, TX USA).

#### Sample size

This study aimed to invite 1500 eligible patients to participate. Based on a survey consent and completion rate of 85%, this would provide 1275 respondents. Assuming a design effect due to clustering of patients within general practice of 1.2, an effective sample size of approximately 1000 would be obtained. This sample size was estimated to allow prevalence estimates with 95% CI’s within ± 3% of the point estimate for proportion wanting to lose weight. Estimating that approximately 40% of the sample would report intending to lose weight, this would allow detection of differences in characteristics between patients intending and not intending to lose weight by 9% for binary exploratory variables, with a 5% significance level and 80% power. Of the 40% intending to lose weight (n = 400), 25% (n = 100) would be obese, 35% would be overweight (*n =* 140) and 40% would be normal weight (n = 160). This would allow the prevalence estimates for reasons for weight loss and preference for assistance with losing weight to be reported with 95% CI within ± 5% of the point estimate within these BMI categories.

## Results

Overall, 2252 patients were invited to complete this survey. Of those, 352 (15%) were ineligible to participate due to the following reasons: less than 18 years of age (*n =* 156); did not feel well enough to complete survey (*n =* 32); could not understand English sufficiently to complete survey, (*n =* 14) had visual impairment (*n =* 7) and other unspecified reasons (*n =* 143). Of those eligible, 1620 (85%) consented to participate in the study and 1,343 patients completed the relevant questions. Almost 3% were excluded (*n =* 37) as they were underweight and results from 1,306 patients are reported. There were no significant differences in the sex of those who consented (39% male) and did not consent (40% male) (χ^2^ = 0.1040; df = 1; p = 0.747). Of the 1306 patients, 35% (*n* = 461) were overweight and 23% (*n =* 299) were obese. There were several differences in characteristics by BMI category in terms of presence of chronic conditions, sex, age and lifestyle risk factors (see Table 1).

**Table 1:** Demographics characteristics of normal weight, overweight and obese general practice patients included in the study

**Characteristic**	**Normal weight**	**Overweight**	**Obese**		**Design based degrees of freedom**	
	**(*n*** **= 546)**	**(*n =*** **461)**	**(*n =*** **299)**			**p-value**
	**n(%)**	**n(%)**	**n(%)**	**Test statistic**		
**Age (yrs)**				5.4	(4.5, 49)	>0.001
18 – 24	47 (8.6)	18 (3.9)	11 (3.7)			
25 – 44	162 (30)	117 (25)	71 (24)			
45 - 64	169 (31)	172 (37)	122 (41)			
≥ 65	168 (31)	154 (33)	95 (32)			
n(%) female	378 (69)	233 (51)	186 (62)	11	(1.6, 18)	0.001
n(%) Caucasian	473 (87)	403 (87)	247 (83)	1.5	(1.8, 20)	0.2
**Number of times previously seen GP in last 12 months**				4.5	(3.5, 38)	0.007
0 – 3	268 (52)	203 (47)	90 (34)			
4 – 6	139 (27)	119 (28)	81 (31)			
7 – 10	51 (9.9)	54 (13)	36 (14)			
≥ 10	58 (11)	43 (13)	57 (22)			
**Level of education (n = 1197)**^**a**^				1.7	(3.4, 38)	0.2
Completed HSC and below	212 (42)	196 (45)	136 (48)			
TAFE or Diploma	87 (17)	66 (15)	53 (19)			
University	165 (32)	134 (31)	67 (24)			
Postgraduate	42 (8.1)	27 (6.2)	12 (4.3)			
**n(%) insufficient level of physical activity**	230 (42)	212 (46)	188 (63)	22	(1.5, 16)	>0.001
**n(%) smokers**	57 (10)	49 (11)	33 (11)	0.03	(1.8, 19)	0.9
**n(%) PHQ >10**	70 (13)	57 (12)	69 (23)	9.9	(1.6, 18)	0.002
**n(%) with heart disease**	47 (8.6)	54 (12)	38 (13)	2.8	(1.7, 18)	0.1
**n(%) with chronic pain**	33 (6.0)	35 (7.6)	49 (16)	24	(1.7, 19)	>0.001
**n(%) with high blood pressure**	112 (21)	162 (35)	154 (52)	50	(1.7, 19)	>0.001
**n(%) with high cholesterol**	87 (16)	133 (29)	95 (32)	19	(1.9, 21)	>0.001
**n(%) with type 2 diabetes**	18 (3.3)	26 (5.6)	50 (17)	16	(2.0, 22)	>0.001

^a^number less than total due to incomplete surveys.

HSC: High school certificate (equivalent to completion of high school); TAFE: Technical and Further Education (equivalent to technical certificate).

### Proportion intending to lose weight

More than half (*n* = 731, 56% [95% CI: 49%, 63%]) the participants reported intending to lose weight, 38 (3.0% [95% CI: 2.0%, 4.3%]) intended to put on weight and 36% [95% CI: 49%, 44%] (*n* = 476) did not intend to change their weight in the next six months. Five percent (*n =* 61) were unsure about whether they intended to change their weight.

### Clinical and demographic associates with intending to lose weight

A number of characteristics were associated with intending to lose weight in the next 6 months (see Table 2). Being obese was associated with 20 times the odds of intending to lose weight. Those aged ≥65 years had significantly lower odds of intending to lose weight than those aged 18–24 years. Females or those with a diploma or technical level of education and above were at significantly increased odds of intending to lose weight in the next 6 months compared to those with who had completed high school and below. Having high cholesterol was also significantly associated with intending to lose weight in the next six months. A score of 10 or more on the PHQ-9 was associated with 1.8 times increased odds of intending to lose weight and this was approaching significance (p = 0.05).

**Table 2:** Adjusted odds ratio for demographic and clinical characteristics associated with general practice patients’ intention to lose weight in the next 6 months (n = 1197)

**Variables**	**n(%) not intending to lose weight**	**n(%) intending to lose weight**	**Adjusted odds ratio**	**95% CI**	p-value^*^
**BMI category**					
Normal weight	340 (67)	166 (33)	1.0		
Overweight	145 (34)	278 (66)	6.6	[4.8, 9.3]	<0.001*
Obese	37 (14)	231 (86)	20	[9.0, 45]	<0.001*
**Sex**					
Male	269 (55)	215 (45)	1.0		
Female	294 (38)	474 (62)	3.9	[2.9, 5.1]	<0.001*
**Scores on PHQ-9**					
<10	506 (47)	563 (53)	1.0		
≥10	57 (31)	127 (69)	1.8	[1.0, 3.3]	0.05
**Age**					
18-24	46 (55)	38 (45)	1.0		
25-44	127 (37)	218 (63)	1.4	[0.7, 2.8]	0.4
45-64	173 (40)	264 (60)	0.8	[0.8, 1.5]	0.5
65+	217 (56)	170 (44)	0.4	[0.2, 0.8]	0.009*
**Presence of chronic pain**					
No	522 (46)	625 (54)	1.0		
Yes	41 (29)	65 (61)	0.8	[0.5, 1.3]	0.3
**Presence of high cholesterol**					
No	455 (47)	498 (52)	1.0		
Yes	108 (36)	192 (64)	1.6	[1.3, 2.1]	0.001*
**Presence of high blood pressure**					
No	414 (49)	437 (51)	1.0		
Yes	149 (37)	253 (63)	1.8	[1.0, 2.5]	0.1
**Education (*n = 1147)***^**a**^					
High school education and below	293 (51)	281 (49)	1.0		
TAFE/Diploma	88 (41)	128 (59)	1.9	[1.3, 2.8]	0.005*
Tertiary education	150 (39)	230 (61)	2.0	[1.4, 2.9]	0.001*
Postgraduate	32 (39)	51 (61)	2.5	[1.3, 5.1]	0.01*

PHQ-9: Patient Health Questionnaire-9; BMI: Body Mass Index; TAFE: Technical and Further Education (equivalent to technical certificate).

* p-value <0.05 indicates significant variables in multiple logistic regression for intending to lose weight in next six months.

^a^number less than total due to incomplete surveys.

### Reason for intending to lose weight

The most endorsed top reason for intending to lose weight were for “health” (58%, [95% CI 53, 62]), wanting to “achieve ideal weight” (10%, [95% CI 8.1, 13]) and to “improve physical fitness” (9.7% [95% CI 7.2, 13]). “Health” was the top ranked reason for wanting to lose weight among participants in all BMI categories (see Table 3). This was particularly so for obese participants, with 72% of obese indicating health as a top reason for intending to lose weight, compared to 57% of overweight and 38% of normal weight ( F(1.8, 20): 30, p < 0.001). All other reasons were endorsed by less than 10% of obese patients as top reasons for intending to lose weight. More than 10% endorsed to “achieve ideal weight” as a top reason for wanting to lose weight in the overweight group. In the normal weight group, more than 10% reported that “achieve ideal weight”, “increase fitness” and “improve appearance” as top reasons for intending to lose weight.

**Table 3:** Top ranked and ranked within top three reasons for wanting to lose weight in normal weight, overweight and obese general practice patients intending to change weight in next six months

**Reason for intending to lose weight**	Normal weight (n = 176)*				Overweight (n = 299)*				Obese (n = 253)*			
	**Top reason**		**Within top 3 reasons**		**Top reason**		**Within top 3 reasons**		**Top reason**		**Within top 3 reasons**	
	***n *** **(%)**	**[95% CI]**	***n (%)***	**[95% CI]**	***n (%)***	**[95% CI]**	***n (%)***	**[95% CI]**	***n *** **(%)**	**[95% CI]**	***n *** **(%)**	**[95% CI]**
Health	66 (38)	[30, 46]	76 (43)	[35, 52]	168 (57)	[49, 64]	204 (69)	[62, 75]	182 (72)	[65, 79]	208 (83)	[75, 89]
Achieve ideal weight	23 (13)	[9.2, 18]	83 (47)	[40, 54]	36 (12)	[8.2, 17]	130 (43)	[37, 51]	15 (5.9)	[4.0, 8.8]	119 (47)	[40, 54]
Increase fitness	24 (14)	[10], [18]	83 (47)	[38, 56]	30 (10)	[6.5, 15]	118 (40)	[33, 48]	16 (6.3)	[3.1, 12]	106 (42)	[39, 46]
Improve appearance	33 (19)	[14, 25]	79 (45)	[38, 52]	24 (8.0)	[5.1, 12]	62 (25)	[27, 37]	9 (3.6)	[1.7, 7.2]	62 (25)	[18], [32]
Feel better	2 (1.1)	[0.3, 4.8]	11 (6.3)	[2.5, 15]	13 (4.4)	[2.1, 8.9]	49 (16)	[11, 25]	20 (7.9)	[5.3, 12]	97 (38)	[31, 47]
Currently overweight	13 (7.4)	[3.5, 15]	49 (28)	[21, 36]	16 (5.4)	[2.9, 9.6]	46 (15)	[11, 20]	3 (1.2)	[0.4, 3.3]	28 (11)	[6.8, 17]
Fit into old clothes	3 (1.7)	[0.5, 5.3]	29 (16)	[11, 24]	7 (2.3)	[1.0. 5.4]	34 (11)	[7.7, 16]	2 (0.8)	[0.2, 3.2]	15 (5.9)	[2.8, 12]
Increase confidence	12 (6.8)	[4.2, 11]	37 (21)	[16, 26]	4 (1.3)	[5.1, 12]	35 (12)	[7.5, 18]	4 (1.6)	[1.7, 7.2]	29 (11)	[8.0, 16]

*Not equal to total intending to lose weight due to incomplete surveys.

Similarly, when the top three ranked reasons for losing weight were examined, “health”, “achieve ideal weight” and “increase fitness” were the most frequently endorsed by both overweight and obese participants and “increase fitness”, “achieve ideal weight” and “improve appearance” most frequently reported by those in the normal weight group. Also worth noting is that almost one third (28%) of the normal weight participants indicated “currently overweight” as one of their top three reasons for wanting to lose weight.

### Preferred personnel to assist with weight management

Of those who intended to change their weight in the next six months, 66% [95% CI 60, 71] would like help from one of the personnel. More than half of those overweight (61%) [95% CI 53, 70 ] or obese (74%) [95% CI 68, 79] reported wanting help from one of the listed personnel. The preferred person to help with changing overweight and obese patients weight were the dietitian, GP and exercise physiologist (see Table 4).

**Table 4:** Top ranked personnel to assist overweight and obese general practice patients who intend to lose weight in the next six months with managing their weight

**Most preferred person to assist with weight management**	**Overweight (n = 293)**^**a**^			**Obese (n = 251)**^**a**^			**Design based degrees of freedom**	**Test statistic**	**p-value**
	***n***	**%**	**95% CI**	***n***	**%**	**[95% CI]**			
Dietitian	75	26	[21, 31]	83	33	[30, 36]	(1.8, 20)	2.9	0.08
General practitioner	47	16	[12, 22]	42	17	[12, 23]	(1.8, 19)	1.6	0.2
Exercise physiologist	39	13	[8.7, 20]	36	14	[10, 20]	(1.3, 15)	0.2	0.7
Psychologist	7	2.4	[1.1, 5.2]	10	4	[2.2, 7.1]	(1.4, 16)	1.7	0.2
General practice nurse	1	0.3	[0, 3.1]	4	1.6	[0, 3.9]	(1.9, 21)	2.8	0.09
Surgeon	0	0	n/a	2	0.8	[0.2, 4.2]	(1.1, 12)	0.5	0.5
Weight loss consultant	9	3.1	[1.3, 7.0]	9	3.6	[1.6, 8.0]	(2.0, 22)	1.2	0.3
None of the above	114	39	[36, 49]	66	26	[21, 32]	(1.6, 18)	5.9	0.015*

*Not equal to total intending to lose weight due to incomplete surveys.

^a^ Total percentage not equal to 100 due to rounding up of figures.

### Acceptability of support delivered via different modes

The majority of participants would be willing to accept weight management support delivered via telephone (almost 90% for both overweight and obese categories – see Table 5). Email and SMS were less well received, with less than half of the overweight or obese patients indicating they would be willing to accept support delivered via these mediums. More than half the patients indicated not having access to smart phones or table devices (57%).

**Table 5:** Proportion of overweight or obese general practice patients intending to lose weight in the next six months who report that they would be willing to accept weight support services delivered via different mediums

**Delivery of weight support services**	**% that have access**	**Overweight (n = 300)**			**Obese (n = 255)**			**Test statistic**	**Design based degrees of freedom**	**p-value**
		***n***	**%**^**a**^	**95% CI**	***n***	**%**^**a**^	**95% CI**			
Telephone	100	265	89	[85, 91]	223	88	[77, 94]	0.2	(2.2, 24)	0.8
Email	85	136	45	[40, 41]	105	41	[36, 47]	1.5	(2.8, 31)	0.2
Short messaging service (SMS)	93	128	43	[34, 52]	94	37	[29, 46]	1.1	(2.8, 30)	0.4
Online chat room	83	104	35	[31, 39]	69	27	[24, 30]	2.2	(2.4, 27)	0.1
Smart phone /tablet application	43	80	27	[23, 30]	68	27	[21, 34]	0.5	(2.4, 26)	0.6

*Not equal to total intending to lose weight due to incomplete surveys.

^a^ Percentages were of all intending to lose weight that report being willing to accept support via these mediums.

## Discussion

Despite the potential benefits of using general practice for interventions targeting overweight and obesity, this study is one of few to describe the demographic associates of patients who report intending to lose weight and the acceptability of weight management interventions delivered via different modes in Australian general practice, to our knowledge. While a large proportion of overweight or obese general practice patients report previously trying to lose weight [14,25], the current literature provides little information on the weight management preferences of these patients. Our study found that females, those with high cholesterol, or those with higher level of education had increased odds of intending to lose weight in the next six months. Overweight and obese patients reported that the most preferred person to help them with losing weight was the dietitian and GP and almost all were willing to accept weight management assistance delivered via telephone.

Being overweight, obese, female and reporting higher levels of education were significantly associated with intentions to lose weight in the next six months. These findings are similar to other research examining associates of those previously trying to lose weight and provide an indication of which patients GP’s may be able to initiate weight management discussions with [6,7].

Although intentional weight loss is associated with improved outcomes in those with type 2 diabetes and high blood pressure [26,27], those with these conditions did not have significantly higher odds of intending to lose weight in the next six months. This may be due to the relatively small proportion in the sample that reported having type 2 diabetes (7%). Of the examined weight-related chronic conditions, only presence of high cholesterol was significantly associated with intentions to lose weight. It is possible that patients are not fully aware of the specific benefits of weight loss in improving outcomes such as blood pressure and glycaemic control. Patients may perceive a more direct link between high cholesterol and diet or weight. A qualitative study amongst general practice patients reported that most participants acknowledged an association between diet (especially fat) and high cholesterol and that patients perceived having high cholesterol to be associated with presence of overweight [28]. The link between weight loss or diet and improvements to blood pressure or glucose levels may need to be more clearly communicated to patients either via GPs or through public health messages regarding healthy weight.

All patients wanting to lose weight in the next six months reported health as the top reason. This is consistent with other studies where [29] health reasons were the main motivating factors for attempting weight loss among overweight or obese people [10,12]. Although weight loss in those already in the healthy weight range does not provide increased health benefits, those in the normal weight group similarly indicated that their top reason for intending to lose weight was for health. As those consenting to this survey were asked a variety of screening and health questions, this may have affected participants’ reporting and made it more likely for them to report ‘health’ as a reason for intending to lose weight. Frequent attendees to general practice care may also be more health conscious and thus may have been more likely to choose ‘health’ as a reason for intending to lose weight.

In contrast to other studies involving participants enrolled in weight loss trials [10,12], improving appearance was not endorsed as one of the top reasons for intending to lose weight in those overweight or obese. In the current sample, achieving ideal weight and increasing physical fitness were more frequently endorsed reasons for intention to lose weight than improved appearance. This indicates that reasons for intending to lose weight may differ between general practice patients and those enrolled in weight loss trials.

Overall, 66% of those intending to lose weight indicated that they wanted professional help to do so. The majority rated the dietitian as the preferred person to assist with weight management, followed by GPs and exercise trainers. A previous study identified that patients favoured GP advice compared to dietitian referral [25], while Tham and colleagues found that the GP was rated fourth in the list of ideal person nominated to help with weight loss, after personal trainer, dietitian and weight loss consultant [30]. These discrepancies in findings could be attributed to the differences in age range of the included patients [30] or differences in wording of survey items. It is likely that patients’ preferred personnel for assistance with weight loss was influenced by the type of assistance they expect to receive from these personnel. For example, those who indicate wanting help from a dietitian may like assistance with planning their meals or dietary advice. While the specific content area that patients would like help with was not examined in the current study, a previous study reported that 80% of Australian general practice patients rated advice on healthy eating and physical activity as useful or very useful for weight loss [20].

Our finding that the dietitian and GP are the preferred personnel for providing assistance for weight loss is encouraging. A randomised controlled trial previously demonstrated that dietitian advice in conjunction with brief advice from a GP is effective in producing clinically significant weight loss at six months follow up compared to usual care [31]. Additionally, findings from a systematic review indicate that non-physician delivered counselling with regular GP review is effective in producing weight loss [15]. The involvement of exercise physiologists is also likely to be useful in assisting patients with undertaking physical activity. However, longer term, rigorous evaluations of the involvement of exercise physiologists, dietitians and GPs in delivery of weight loss interventions is needed to confirm this.

Almost 90% of overweight or obese patients indicated willingness to accept support with weight management via telephone. This is in line with a previous study in one Australian state, which found that 87% of participants considered it acceptable for a health service to contact people by telephone to assist them with losing weight, eating healthily and being more physically active [32]. The high acceptability of telephone-delivered support may be due to increased familiarity with this mode, as all participants except one had access to a telephone. Patients may also prefer telephone contact to other modes of delivery, as it involves direct interaction with another person and may provide a more ‘personal touch’. Coupled with findings that telephone-delivered interventions are effective in changing participant’s physical activity levels and dietary intake [16,33], future weight loss interventions in this setting should incorporate telephone contact as method of providing patients with support to lose weight. While more cost-effective than face to face or telephone contact, a lower proportion of patients indicated that they would be willing to accept support via SMS, chat group or email. Some potential reasons for this may be dislike of technology or unlikely to open, read or act on it [32]. Only 27% of overweight or obese patients intending to lose weight indicated being willing to accept support delivered via smart phone or tablet applications. With more than 50% of patients indicating having no access to a smart phone or tablet device, interventions utilising these devices need to take into account potential access and cost barriers.

### Limitations

Findings from this study need to be interpreted in light of the following limitations. Social desirability bias may have led to a higher proportion reporting intending to lose weight in the next six months and indicating health as their top reason for wanting to lose weight. Additionally, participants were required to choose their responses from within pre-specified reasons. Only three participants endorsed “other” as a top reason for wanting to lose weight, suggesting response options were fairly comprehensive. There may have been an overlap between the reasons for wanting to lose weight presented in the survey; however, asking patients to rank the reasons in order of importance provided an indication of how weight loss discussions can be framed so that it is most relevant to these patients. The study also used self-reported weight and height to calculate BMI. Previous research in a subsample of participants in the study identified high overall agreement between self-reported weight and height, although substantial variation in individual reporting was identified [34]. It is likely that the current findings are not generalisable to the general population as health concerns may be more salient to those presenting for general practice care. While preferences for management with weight assistance may vary by sex and age, we were unable to explore this due to small number of patients within each response option. This study however provides valuable information regarding the preferences of general practice patients and the ways in which GPs can best assist their overweight or obese patients with losing weight.

## Conclusions

Those overweight, obese, younger, females, with a level of education of trade certificate and above and have high cholesterol had higher odds of intending to lose weight in the next six month. The high rates of overweight and obese patients intending to lose weight and that “health” was the top reason for wanting to lose weight confirm that there is substantial opportunity for weight loss discussions to be initiated by GPs in context of weight-related conditions. With over 70% of obese patients expressing a preference for help to lose weight, the involvement of dietitians and exercise physiologists may facilitate the provision of intensive weight management counselling without putting additional burden on GPs. Additionally, intervention delivery via telephone is a promising tool for weight management in this setting.

## Competing interests

The authors declare that they have no competing interest.

## Authors’ contributions

SLY was involved in study and questionnaire development, data analysis, interpretation of results and drafting of manuscript. MC and CDE participated in the study design, data analysis, interpretation and drafting of the manuscript. RSF was involved in overall study design, interpretation of results and drafting of manuscript. All authors read and approved the final manuscript.

## Pre-publication history

The pre-publication history for this paper can be accessed here:

http://www.biomedcentral.com/1471-2296/14/187/prepub

## Supplementary Material

Additional file 1 Health risk assessment survey. Click here for file
