# Supplementary material for: A cross-sectional study assessing Australian general practice patients’ intention, reasons and preferences for assistance with losing weight
Source: BMC Fam Pract. 2013 Dec 10;14:187. doi: 10.1186/1471-2296-14-187 (PMC4029270; doi:10.1186/1471-2296-14-187)
Supplement: Additional file 1 — Health risk assessment survey. [file 1471-2296-14-187-S1.doc]

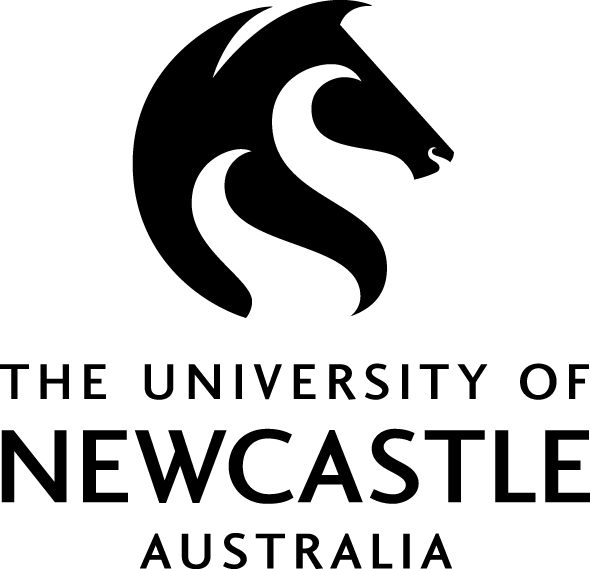

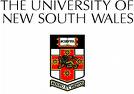

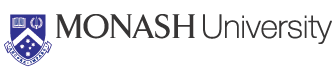


Thank you for your interest in participating in this research project. The information you provide will help us determine whether the touchscreen computer questionnaire is useful in providing us with information regarding your health. Touch the ‘NEXT’ button on the bottom right hand corner of the screen to indicate that you are willing to participate in this study. Please also retain the hard copy version of your information statement for future reference.

Please touch the ‘NEXT’ button when you are ready to commence the survey.

**Insert participant ID**

*Please insert 6-digit ID and then touch ‘NEXT’*

**Module 1: Your background**

**1. Are you**

*Please touch your response and then touch ‘NEXT’*

1= Male

2= Female

**2. What is your age?**

*Please touch your response and then touch ‘NEXT’*

1= 18-24 years

2= 25-29 years

3= 30-34 years

4= 35-39 years

5= 40-44 years

6= 45-49 years

7= 50-54 years

8= 55-59 years

9= 60-64 years

10= 65-69 years

11= More than 70 years

3**. Your ethnic background is:**

*Please touch your response and then touch ‘NEXT’*

1= Aboriginal or Torres Strait Islander

2= Maori and other Pacific Islander

3= South Asian

4= Caucasian (i.e. White)

5= None of the above

**4. Who are you expecting to see today?**

*Please touch your response and then touch the ‘NEXT’ button on the right hand corner.*

1= My usual doctor

2= Not my usual doctor

3= Practice nurse

4= Don’t know

If 1 and 2 is selected for Q4

**5a. Approximately, how many times have you seen this GP in the last 12 months?**

a = 0

b= 1

c = 2

d = 3

e = 4

f = 5

g = 6

h = 7

i = 8

j = 9

k= 10

l= More than 10

**Module 2 : Personal health history**

The following questions are about your health.

**1. Have you ever been told by a doctor or nurse that you have any of these conditions?**

*Please touch all that apply and then touch ‘NEXT’*

1 = High blood pressure

2 = High cholesterol

3 = Heart problems (i.e. blocked arteries, heart attack)

4 = Diabetes (or high blood sugar) Go to 1a

5 = Kidney disease

6 = Depression

7 = Stroke

8 = Chronic pain

9 = None of the above

**1a. What type of diabetes were you told you had?**

*Please touch your response and then touch ‘NEXT’*

1= Type 1 (Usually starts in childhood, needs daily insulin injection)

2= Type 2 (Usually starts in adulthood, may not need insulin injection)

3= Gestational (occurs during pregnancy)

4= Pre-diabetes (high blood sugar, this includes impaired glucose tolerance and impaired fasting glucose)

**2. Which of the following best describes your smoking status? This includes cigarettes, cigars and pipes.**

*Please touch your response and then touch ‘NEXT’*

1= I smoke daily

2= I smoke occasionally

3= I don’t smoke now, but I used to

4= I have tried if a few times but never smoked regularly

5= I have never smoked

3**. Were any of your close BLOOD relatives (parents, brothers, sisters, or children) ever diagnosed with heart disease?**

*Please touch your response and then touch ‘NEXT’*

1= Yes

2= No

3= Not sure

*If 1 selected for Q3*

**3a. Were they diagnosed at 60 years of age or younger?**

*Please touch your response and then touch ‘NEXT’*

1= Yes

2= No

3= Not sure

**Module 3a: Screening for CVD- related risk**

This information will be used to answer the next question

The following questions are regarding some tests that you may have had to detect risk factors related to heart disease.

*Please read the text below and touch ‘NEXT’ when you have finished reading*

CHOLESTEROL TESTING

*Cholesterol info screen*

To test for cholesterol levels, a blood sample would have been drawn from your arm. Your doctor may have asked you to fast for nine to 12 hours.

For those with average risk (as determined by the algorithm);

**1a. When did you last have your cholesterol tested?**

*Please touch your response and then touch ‘NEXT’*

1= Never

2= In the last 6 years (between 2004 and now)

3= More than 6 years ago

4= Not sure

For those at increased risk (as determined by the algorithm);

**1b. When did you last have your cholesterol tested?**

*Please touch your response and then touch ‘NEXT’*

1= Never

2= In the last 3 years (between 2007 and now)

3= More than 3 years ago

4= Not sure

For those with high risk (as determined by the algorithm),

**1c. When did you last have your cholesterol tested?**

*Please touch your response and then touch ‘NEXT’*

1= Never

2= In the last 2 years (between 2008 and now)

3= More than 2 years

4= Not sure

For those who have high cholesterol (Module 2, Q1, 2 is selected),

**1d. Which best describes how often you have your cholesterol tested?**

*Please touch your response and then touch ‘NEXT’*

1= Never had my cholesterol tested

2= About once every 12 months

3= More than once every 12 months

4= Less often than every 12 months

5= Not sure

BLOOD PRESSURE (FOR ALL PARTICIPANTS)

The information will be used to answer the next question.

To test for **high blood pressure**, a rubber cuff is wrapped around your upper arm and then inflated to measure your blood pressure.

*Please read the text below and then touch ‘NEXT when you have finished reading.*

For those with average risk (as determined by the algorithm):

**2a. When did you last have your blood pressure checked?**

*Please touch your response and then touch ‘NEXT’*

1= Never

2= In the last 3 years (between 2007 and now)

3= More than 3 years ago

4= Not sure

For those with increased risk (as determined by the algorithm),;

2b. **When did you last have your blood pressure checked?**

*Please touch your response and then touch ‘NEXT’*

1= Never

2= In the last 2 years (between 2008 and now)

3= More than 2 years ago

4= Not sure

For those with high risk (as determined by the algorithm);

**2c. When did you last have your blood pressure checked?**

*Please touch your response and then touch ‘NEXT’*

1= Never

2= In the last 12 months

3= More than 12 months ago

4= Not sure

For those who have high blood pressure (Module 2, Q1, 1 is selected),

**2d. Which best describes how often you have your blood pressure checked?**

*Please touch your response and then touch ‘NEXT’*

1= Never had my blood pressure checked

2= About once every 12 months

3= More than once every 12 months

4= Less often than every 12 months

5= Not sure

TYPE 2- DIABETES TESTING

The information will be used to answer the next question.

To test for blood sugars level, a blood sample would have been drawn from your arm. Your doctor would have asked you to fast and drink only water for nine to 12 hours before your blood test.

*Please read the text below and then touch ‘NEXT’ when you have finished reading.*

**3a.** **When was the last time a GP or nurse measured your blood sugar level?**

*Please touch your response and then touch ‘NEXT’*

1= Never

2= In the last 4 years (between 2006 and now)

3= More than 4 years ago

4= Not sure

For those with existing type 2 diabetes (Module 2, Q1, 4 is selected);

The HbA1c test is a blood test that measures if your blood sugar is under control.

**3b. Which best describes how often you have an HbA1c test?**

*Please touch your response and then touch ‘NEXT’*

1= Never had an HbA1c test

2= About once every 12 months

3= More than once every 12 months

4= Less often than every 12 months

5= Not sure

**Module 3b : Cancer screening**

This section is about tests that you may have had to screen for cancer or things you may have done to reduce your risk of cancer.

**1. Have you ever had cancer?**

*Please touch your response and then touch ‘NEXT’*

1= Yes go to 1a/1b

2= No go to 2

**For males,**

**1a. Which type of cancer did you have? (*Pick all that apply)***

*Please touch all that apply and then touch ‘NEXT’*

1= Melanoma

2 = Bowel / Colorectal

3= Prostate

4= Lung

5= Non-Hodgkin’s lymphoma

6= Brain

7= Head and neck

8= Other

**For females,**

**1b. Which type of cancer did you have? (*Pick all that apply)***

*Please touch all that apply and then touch ‘NEXT’*

1= Melanoma

2 = Cervical

3 = Breast

4 = Bowel / Colorectal

5= Lung

6= Non-Hodgkin’s lymphoma

7= Brain

8= Head and neck

9= Other

**2. Have any of your BLOOD relatives (parents, brothers, sisters, children, aunties, uncles grandparents or grandchildren) ever been diagnosed with any of the following cancers?** *Please touch all that apply and then touch ‘NEXT’*

1 = Breast

2 = Bowel/Colorectal

3 = Ovarian

4= Melanoma

5 = None of the above

If participant has history of melanoma (Q1b= 1 or Q1c=1) or family history of melanoma (Q2=4) is selected

**3. When was the last time your doctor checked all or most of your skin for moles or changes in your skin?**

*Please touch your response and then touch ‘NEXT’*

1= Never

2= In the last 2 years (between 2008 and now)

3= More than 2 years ago

4= Not sure

**Faecal Occult Blood Test (FOBT)**

For a FOBT, you would have been asked to provide samples of faeces. The samples would have been tested for tiny amounts of blood.

**For those aged >50,**

**4. When was the last time you had a FOBT?**

*Please touch your response and then touch ‘NEXT’*

1= Never had a FOBT

2= In the last 3 years (between 2007 and now)

3= More than 3 years ago

4= Not sure

**5. Have you ever received an FOBT kit in the mail as part of an invitation to participate in the National Bowel Cancer Screening Program?**

*Please touch your response and then touch ‘NEXT’*

1= Yes

2= No

3= Not sure

*All females aged 40-69 without a personal history of breast cancer;*

**Mammogram**

This is a test for the early detection of breast cancer. An x-ray is taken of your breast by a machine that presses against your breast while the picture is taken.

**6. When did you last have a mammogram?**

*Please touch your response and then touch the ‘NEXT’ button on the right hand corner.*

1= I have never had a mammogram

2= Within the last 3 years (between 2007 and now).

3= More than 3 years ago.

4= Not sure

*For females only,*

A total **hysterectomy** is an operation in which a woman’s uterus (or womb) is completely removed.

**7. Have you had a total hysterectomy?**

1= Yes go to depression section

2= No go to Q8

For all females, who have not had a total hysterectomy (Q7=2),

**Pap smear test**

This is a test for the early detection of cancer of the cervix. This test involves a doctor taking a few cells from the cervix and sending them to a laboratory to be tested.

**8. When did you last have a Pap smear test?**

1= Never

2= In the last 3 years (between 2007 and now)

3= More than 3 years ago

4= Not sure

**Module 4 : Lifestyle Risk factors**

The following questions are about lifestyle factors and habits that can affect your health.

**1. How often do you usually have a drink containing alcohol?**

*Please touch your response and then touch the ‘NEXT’ button on the right hand corner.*

1= Never

2=Monthly or less

3=2-4 times per month (once a week or once every 2 weeks)

4=2-3 times per week

5=4 or more times per week

**
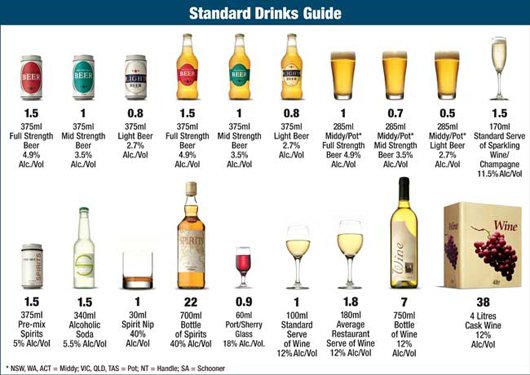
**

**2. On a typical day that you have an alcoholic drink, how many STANDARD drinks do you usually have?**

**(*Note: One middy/100mls of wine = 1 standard drink***

***One schooner/375ml premixed can = 1.5 standard drinks, One bottle of wine = 7 standard drinks)***

_______

**3. How often do you have 4 or more drinks on one occasion?**

*Please touch your response and then touch the ‘NEXT’ button on the right hand corner.*

1= Never

2= Less than monthly

3= Monthly

4= Weekly

5= Daily or almost daily

**4. As a rule, do you do at least half an hour of moderate or vigorous exercise (such as walking or a sport) on five or more days a week?**

*Please touch your response and then touch the ‘NEXT’ button on the right hand corner.*

1= Yes

2= No

3= Unsure

**5** **Please enter your weight in kilograms. If you only know your weight in stones, please press NEXT.**

*Please insert using number pad provided. Use the decimal point if needed.*

If none answered for Q5,

**5a. Please enter your weight in STONE.**

*Please insert using number pad provided. Use the decimal point if needed.*

**6. How tall are you without shoes?**

*Please give your best estimate.*

🞎 feet 🞎🞎 inches

If none answered for Q6,

**6a. Please enter your height in centimetres (CM)**

*Please insert using number pad provided. Use the decimal point if needed.*

**Module 5c: Weight changes**

**1.Have you tried to change your weight in the past 12 months?**

1= Yes, have tried to lose weight

2= Yes, have tried to gain weight

3= No

4= Not sure

***If Module 5c, Q1, 2 is selected:***

**2. What strategies have you used to gain weight in the past 12 months? *Select all that apply***

1= Prescription medication

2= Over the counter supplements

3= Changed my diet

4= Increased exercise

5= Other

*This information is useful for answering the next question. Please touch "NEXT" when you are ready to answer the next question.*

**Professional weight loss centre programs**

This refers to weight loss programs where you make diet and exercise changes with assistance from a weight loss consultant. Example of weight loss centres are Jenny Craig and Tony Ferguson.

**Prescription medication**

Your doctor will have given you a script for some medication that you take to help you lose weight. Examples of prescription medication are Duromine or Reductil.

**Surgery**

This refers to surgical procedures such as gastric banding or gastric bypass.

**Over-the counter supplements**

This includes all herbal and non-herbal weight loss supplements that you can get over the counter. Examples include guarana, weight loss tea or weight loss pills.

**Increased exercise**

This refers to any intentional attempts to increase exercise levels to lose weight.

**Changed diet**

This refers to any intentional changes to your diet to lose weight.

**Consulted a weight loss specialist**

This includes consulting a dietary or exercise specialist including a dietitian or exercise physiologist.

***If Module 5c, Q1, 1 is selected:***

**3. Which strategies have you tried to lose weight in the past 12 months?**

***Select all that apply***

1= Professional weight loss centre program (e.g. Jenny Craig )

2= Prescription medication

3= Surgery

4= Over the counter supplements

5= Increased exercise

5= Changed diet

6= Consulted a weight loss specialist

7= Other

***If Module 5c, Q3, 9 is selected:***

**3a. Please specify what other strategies you used.**

***If Module 5c, 1-5 selected for Q2 or 1-6 selected for Q3;***

**3b. Did you consult your GP before using these weight loss strategies?**

1= Yes

2= No

*This information is useful for answering the next question. Please touch "NEXT" when you are ready to answer the next question.*

**Specialised meal replacements**

Meal replacements are foods that are taken as a substitute for a solid food meal. They can come in the form of drinks, snack bars or frozen meals.

**Low calorie diet**

This is when you restrict your food intake so that you reduce the overall calories consumed.

**Low carbohydrate/High protein diet**

This includes a diet low in carbohydrates (such as rice, bread, pasta) and high in protein (such as meat, eggs, dairy). An example of a low carbohydrate diet is the Atkin’s diet.

**Low fat diet**

This diet includes intentionally cutting down on fat in your diet. Examples of food that are high in fat are high-fat meat, cake or pastries or snack food.

**Detox diet**

Detox diets involve not consuming or attempting to flush out substances that are considered harmful. Examples include restricting certain food that contain colourings or preservatives or taking supplements to induce diarrhoea.

**High fibre diet**

This diet involves intentionally increasing the fibre content of your diet. Some examples of food high in fibre are vegetables, fruit and wholegrain products.

**Celebrity/fad diets**

These diets involve making extreme, rapid changes to food consumption, often recommended in celebrity magazines. Examples include grapefruit diet, cabbage soup diet

***If Module 5c, Q3, 5 is selected:***

**3bc What diets have you tried in the past 12 months?**

*Please touch all that apply*

1= Specialised meal replacements

2= Low calorie diet (Reduced food)

3= Low carbohydrate diet (Atkins diet)

4= Low fat diet

5= Detox diet

6= High fibre diet

7= Celebrity/fad diets

8= Other

***If Module 5c, Q3c, 9 is selected:***

**3b. Please specify what diet you have used in the last 12 months.**

**4a. Did these strategies help you gain weight in the last 12 months?**

1= Yes, gained weight

2= No, weight has not changed

3= No, lost weight

4= Not sure

**5. Are you currently trying to change your weight?**

1= Yes

2= No

Intending to change weight in this question means that you have considered the benefits and costs of changing your weight. You are planning to make the required changes in the next 6 months in order to achieve this.

**4a. Do you intend to change your weight in the next 6 months?**

*Please touch your response and then touch the ‘NEXT’ button on the right hand corner.*

1= Yes, intend to put on weight

2= Yes, intend to lose weight

3= No, do not intend to change weight

4= Not sure

***If yes, want to put on weight:***

**5a. Why do you want to gain weight?**

*Please rank up to 3 in order of importance. Touch the most important reason first, followed by the second and third most important reasons.*

1= For health reasons

2= To increase my physical fitness

3= To increase my confidence

4= To improve my appearance

5= To achieve my ideal weight

6= To feel better

7= I am currently underweight

8= To fit into my old clothes

9= Other

***If yes, want to lose weight:***

**6a. Why do you want to lose weight in the next 6 months?**

*Please rank up to 3 in order of importance. Touch the most important reason first, followed by the second and third most important reasons.*

1= For health reasons

2= To increase my physical fitness

3= To increase my confidence

4= To improve my appearance

5= To achieve my ideal weight

6= To feel better

7= I am currently overweight

8= To fit into my old clothes

9= Other

**If yes want to put on weight/lose weight (Q5),**

**8. Which of the following personnel would you like assistance from to change your weight?**

*Rank up to 3 health professionals you would like help from in order of preference. Touch your first preference, followed by the second and third.*

1= General practitioner

2= Nursing staff

3= Dietitian

4= Psychologist

5= Exercise Physiologist

6= Surgeon

7= Weight loss consultant

8= None of the above

**9. Would you be willing to receive support with managing your weight by:**

*Please select an answer for each row. Please select no access if you do not have regular access to any of the devices*

1. Telephone 1=Yes/ 2= No/ 3= No access
2. Email 1=Yes/ 2= No/ 3= No access
3. Short messaging service (SMS) 1=Yes/ 2= No/ 3= No access
4. Smart phone/tablet application 1=Yes/ 2= No/ 3= No access
5. Online chat 1=Yes/ 2= No/ 3= No access

**Module 6: Quality of Care Module + Other demographics**

**1. Have you been to this clinic before?**

1= Yes

2= No

If yes for 1 is selected;

**2. Please indicate the area(s) of your general practice care at THIS CLINIC that you would have liked improved.**

*Please rank as many as apply in order of importance to you. Touch the most important first.*

1= Management of my physical symptoms

2= Information and communication about my healthcare

3= Emotional support

4= Involvement of and support of my family/friends

5= Being treated compassionately and with dignity

6= Access to healthcare when needed

7= Support to cope with my relationships

8= Assistance with practical concerns (e.g. child care)

9= No improvements in any of these areas needed.

**3. Which of the following best describes the main reason you are visiting the doctor today?**

*Please touch your response and then touch the ‘NEXT’ button on the right hand corner.*

1=For a new problem

2=For an existing or chronic problem

3=For a work-related problem

4=For a medication problem or to get a prescription

5=For a treatment

6=To get the results of tests

7=For a general check-up

8=To get a referral to a specialist

9= To get vaccinated (e.g. flu shot)

10=Other

**4. Why did you choose to come to this general practice?**

*Please choose up to 3 and rank in order of importance. Touch the most important factor 1st, followed by the 2nd and 3rd.*

1= It is close to where I live / work.

2= It is easy to travel to this practice.

3= This practice bulk bills.

4= I am usually able to see the same doctor each time I come.

5= I feel comfortable with the doctors here.

6= It is the only place where I can see a doctor.

7=The reception staff in this practice are very helpful

8= The practice offers after hours consultations

9= I bring other family members to this practice.

10= This practice offers drop-in appointments

11= It is easy to find parking

12= I like the doctors here.

**5. What is the highest level of education you have completed? *Please select only one.***

*Please touch your response and then touch the ‘NEXT’ button on the right hand corner.*

1=Primary school

2=Some high school

3=Year 10 (School certificate)

4= Completed HSC (Year 12)

5= TAFE certificate or Diploma

6= University or other tertiary qualification

7= Postgraduate qualifications (Master or Doctorate)

8 = Other

**6. Do you have:**

**a) A health care card** 1= Yes 2= No

**b) A Veteran Affairs card** 1= Yes 2= No

**c) Private health insurance** 1= Yes 2= No

**d) Pensioner concession card** 1= Yes 2= No

**7. What is the postcode where you live?** _____________
